# Aesthetic assessment of maxillary lateral and canine implant-supported crowns by laypersons and orthodontists

**DOI:** 10.2340/aos.v83.40738

**Published:** 2024-06-12

**Authors:** Nina Sabel, Erika Toft, Erica Johansson, Julia Naoumova

**Affiliations:** a Department of Pediatric Dentistry, Institute of Odontology, Sahlgrenska Academy, University of Gothenburg, Göteborg, Sweden; b Public Dental Service, Region Västra Götaland, Göteborg, Sweden; c Specialist Clinic for Orthodontics, Gothenburg, Public Dental Service, Region Västra Götaland, Göteborg, Sweden; d Department of Orthodontics, Institute of Odontology, Sahlgrenska Academy, University of Gothenburg, Göteborg, Sweden

**Keywords:** Aesthetics, prom, dental appearance, laypeople, orthodontics, implant

## Abstract

**Introduction:**

The objectives of this study were to evaluate how laypersons and orthodontists evaluate and rank aesthetic parameters of an implant-supported crown (ISC) on the canine position (ISC-C) and lateral position (ISC-L).

**Methods:**

A digital survey of 11 cases, 5 ISC-C, 5 ISC-L and 1 control case without ISC, was distributed to 207 laypersons and 296 orthodontists. All cases included one extraoral photograph and three intraoral photographs. The respondents were asked to identify the ISC and to evaluate the aesthetic parameters regarding colour of the implant (CI), shape of the implant (SI) and gingival colour around ISC (GCI). Differences within and between the groups were tested using Chi-2-test and Independent-Samples *t*-test.

**Results:**

All invited laypersons and 184 orthodontists (62% response rate) answered the survey. Orthodontists (89%) more correctly identified the ISC, regardless of its position, than laypersons (50%) (*p* < 0.001). Both laypersons (54%) and orthodontists (23%) rated higher proportions of acceptance of CI, SI and GCI in favour for the ISC-L than ISC-C (laypersons: 40%, orthodontists: 10%) (*p* < 0.001). Assessing each parameter separately, orthodontists rated higher for ISC-L, compared to the ISC-C (*p* < 0.001). In general, laypersons and orthodontist ranked tooth colour (mean, standard deviation [SD]:8.0,1.5 and 9.0, 1.0) and tooth shape (mean, SD: 8.0, 1.7 and 8.8, 1.2) as aesthetically higher than the gingival colour (mean, SD: 7.2, 2.2 and 8.0, 1.7) (*p* > 0.001).

**Conclusion:**

Laypersons and orthodontists consider the ISC-L as aesthetically more preferable, compared to the ISC-C.

## Introduction

Reasons for a missing maxillary lateral or canine may be due to dental agenesis [1, 2] or surgical removal because of impaction [3, 4] or trauma [5]. Treatment of a missing tooth in the aesthetic zone, that is in the maxillary front region, is challenging [6]. Three common, well-qualified treatments for replacing a missing maxillary lateral or canine are orthodontic space opening and placement of a single implant, orthodontic space closure with canine or premolar substitution, and tooth-supported restoration [6–9]. Choosing the most suitable treatment for the individual patient is a complex decision depending on factors such as the patient’s malocclusion, available space, profile, growth pattern, and the characteristics of gingiva and periodontium [7].

Smile is a crucial factor in facial expression [10] and plays an important part in how a person is perceived [11]. There is a strong correlation between smile attractiveness and face attractiveness [12], and self-perception and self-esteem [13]. The aesthetics of the smile is not only determined by teeth, but also by the visible gingiva and the lips framing the mouth [14]. In recent years, the demand for aesthetic dentistry has expanded, driven by an increased consciousness of aesthetics [15]. A lower acceptance of dental appearance is seen among younger people [16], with girls more dissatisfied with their dental appearance than boys [17–19]. Several studies have shown that colour, shape, and marginal gingiva of an implant-supported crown (ISC) are critical parameters affecting the aesthetic outcome [14, 20–23]. Dental asymmetries may also affect the aesthetics of the smile. Asymmetries of the maxillary incisor such as gingival margin height, dental midline shift and wear of the canine cusp were evaluated by orthodontists, prosthodontists, and lay persons. Smiles with asymmetries of the gingival margin height and dental midline shift resulted in a less attractive smile [24]. Incisal asymmetries of the central and lateral incisors, compared to their contralateral tooth, are also considered to give a more unattractive smile [25].

The layperson’s perception of the teeth and smile aesthetics are important to the orthodontist when deciding which treatment will be best for the patient. The interpretation of what is aesthetically pleasing is subjective and varies depending on the individual’s subjective preferences, personal experience, and social environment [26]. Orthodontists are specially trained to pay attention and to assess features that might not be of consideration among the general dentist or laypersons [26, 27]. The threshold for unattractiveness has been reported lower among orthodontist than laypeople regarding smile aesthetics such as: unilateral crown length discrepancies [28], bilateral crown length discrepancies [29], gingival display [29], midline discrepancies [30], diastema [28], buccal corridors [31], tooth shape and colour [32].

The placement of an implant in the aesthetic zone usually requires an interdisciplinary approach with an orthodontist, oral surgeon, or periodontist, together with a prosthodontist, to minimise complications and achieve an optimal aesthetic and functional result. Treatment of replacing the maxillary lateral with an ISC is a well-established aesthetic treatment option when the permanent maxillary lateral is missing. Professional assessment of the aesthetic outcome of ISC anteriorly in the maxilla involves the pink aesthetic score and the white aesthetic score [33, 34]. The subjective evaluations consist how satisfied the patients is about the crown colour and gingival colour, and crown height [7, 35, 36].

Treatment assessment of missing laterals are often compared with orthodontic space closure and ISC evaluated by both laypersons and orthodontists [7, 35, 37, 38]. However, to this date and to the knowledge of the authors, there are no studies exploring the aesthetics of ISC in the canine position and the lateral position. Therefore, the primary aim of the study was to analyse laypersons and orthodontists’ identification skills and evaluation of the aesthetics of an ISC, replacing a maxillary lateral or canine. Furthermore, to describe how laypersons and orthodontists rank the importance of the aesthetic parameters, that is colour, shape, and gingival colour.

The hypotheses were:

1. The ISCs on the lateral position will be more frequently identified and assessed as being more aesthetic appealing than a crown on the canine position, according to laypersons and orthodontists.
2. The gingival colour will be rated by both orthodontists and laypersons as a more acceptable parameter than the crown colour and shape in the cases included in the survey.
3. In general terms, the gingival colour is ranked to be the least important aesthetic parameter.

## Materials and methods

### Subjects

The study protocol was approved by the Swedish Ethical Review Authority (Dnr: 2020- 01826). Patients treated between 2004 and 2006 at the specialist clinic of Orthodontics and Prosthodontics in Borås, Public Dental Service, Region Västra Götaland, Sweden, were recruited to this prospective cohort study. The inclusion criteria were patients between the age of 20 and 25 years, unilateral agenesis of maxillary lateral or canine, extracted maxillary lateral or canine, ISC on the missing/extracted position, and pre-prosthodontic orthodontic treatment with fixed appliance. The exclusion criteria were cleft lip and palate, and craniofacial syndromes.

Eighteen cases met the criteria: 5 patients with ISCs on the canine position (ISC-C) and 13 patients with ISCs on the lateral position (ISC-L). Five out of 13 patients were randomly chosen to match the number of canine cases. In total, 10 patients (7 females and 3 males) were included in the study. In the ISC-C group, four implants were placed on right side and one implant on the left side. The reasons for implant placement were impaction (*n* = 3) and agenesis (*n* = 2). In the ISC-L group, four implants were placed on the right side and one on the left side due to agenesis (*n* = 4) and severe root resorption (*n* = 1). One patient without a single implant, who had undergone orthodontic fixed appliance treatment, was also included. The control case was in the same age-span as the patients in the implant group.

The surgical procedure was performed by two specialists at the Department of Oral and Maxillofacial Surgery in Borås, Public Dental Service, Region Västra Götaland, Sweden. The prosthetic treatments were performed by three specialists at the Specialist Clinic of Prosthodontics in Borås, Public Dental Service, Region Västra Götaland, Sweden.

In three of the cases, fixtures with bone-level external connection fixture were used, and the remaining seven cases had fixtures with tissue-level internal connection. All single-implants were provided with ceramic crowns except one with a metal-ceramic crown. All patients were treated with pre-prosthodontic orthodontic treatment with fixed appliance in one or both jaws, by eight different specialists.

### Method

A digital survey was created of the 11 included patients (5 ISC-C, 5 ISC-L and 1 control case without ISC) who had extraoral and intraoral photographs taken at the end of the treatment: one frontal photo with natural posture and three intraoral photos – front, right and left view. Before the respondents were presented the cases, they were not informed that the patients were treated with orthodontic treatment and had ISC. Each case consisted of four questions, except for the control case, which had one question. On all the questions, one option was possible to choose. The questions were mandatory and response editing was not possible.

For each case, four questions were asked: (1) identification of any artificial tooth, (2) the colour (CI), (3) shape (SI), and (4) gingival colour (GCI) of the ISC compared to the contralateral tooth. The identification of artificial tooth was made viewing the extraoral frontal photograph and the rest of the questions were answered viewing the intraoral photographs. On question 2–4, the ISC and contralateral tooth were marked with a number to point out which teeth should be assessed. A 5-point Likert-scale, with options ‘bad’, ‘less good’, ‘acceptable’, ‘good’ and ‘excellent’, was used. The respondents were also asked to range in general, how important they rank tooth colour, tooth shape, and gingival colour, using a visual analogue (VSA)-scale from 0 to 10. The order of the patient cases in the survey was decided by drawing lots.

The survey was answered by laypersons between the age of 20 and 25 years, who had neither studied nor worked in any dentistry field and were selected according to gender and age from the city centre of Gothenburg, Sweden with a population of approximately 550.000 inhabitants. The questionnaire for laypersons was made in Google Forms and access to the survey was through personal contact or scanning a QR code with a smartphone.

Orthodontists, who were members of the Swedish Orthodontic Society were asked to participate. The survey for the orthodontists consisted of the same patient cases but with some modifications; the orthodontists were not asked to evaluate the aesthetics of the contralateral tooth, and whenever the orthodontist choose the options ‘bad’ or ‘less good’, an additional question with multiple choice options was given. The questionnaire for the orthodontists was made in esMaker and sent by email, including information regarding the study and a link to the digital survey. Three reminder emails were sent during a 1-month period for those who had not replied.

### Sample size calculation

The sample size was calculated on the assumption that there is a 25% difference in acceptable aesthetic rating between laypersons and orthodontists for ISCs on the lateral and canine position (significance level of 0.001), which was based on the article published by Josefsson & Lindsten [7]. The sample size analysis yielded that a minimum of 162 respondents is needed to acquire a power of 90%. This number was increased to 200 for questionnaires with missing replies.

### Statistical analysis

All data were analysed using Statistical Package for the Social Sciences (SPSS) software (version 27). Pearson’s chi-squared (Chi-2) test was used to analyse the answers within laypersons and orthodontists, when also comparing the two groups. Independent-Samples *t*-test were used to find differences in ranking importance of parameter. The differences between groups were tested for significance; differences with probabilities less than 5% (*p* < 0.05) were considered statistically significant.

For statistical analyses, alternatives ‘bad’ and ‘less good’ were grouped and represented as ‘non-acceptance of aesthetics’. Alternatives ‘acceptable’, ‘good’ and ‘excellent’ were grouped and represented as ‘acceptance of aesthetics’.

## Results

The survey was sent to 296 members of the Swedish Orthodontic Society, where 184 responded, resulting in a 62% response rate. Majority (63%) of the orthodontic respondents were female (*n* = 116) and the remaining (37%) were male (*n* = 68).

All 207 laypersons who were invited to participated responded, giving a 100% response rate. Of the laypersons, 51% (*n* = 106) were female and 49% male (*n* = 101).

### Identification of the implant-supported crown

**Figure 1:**
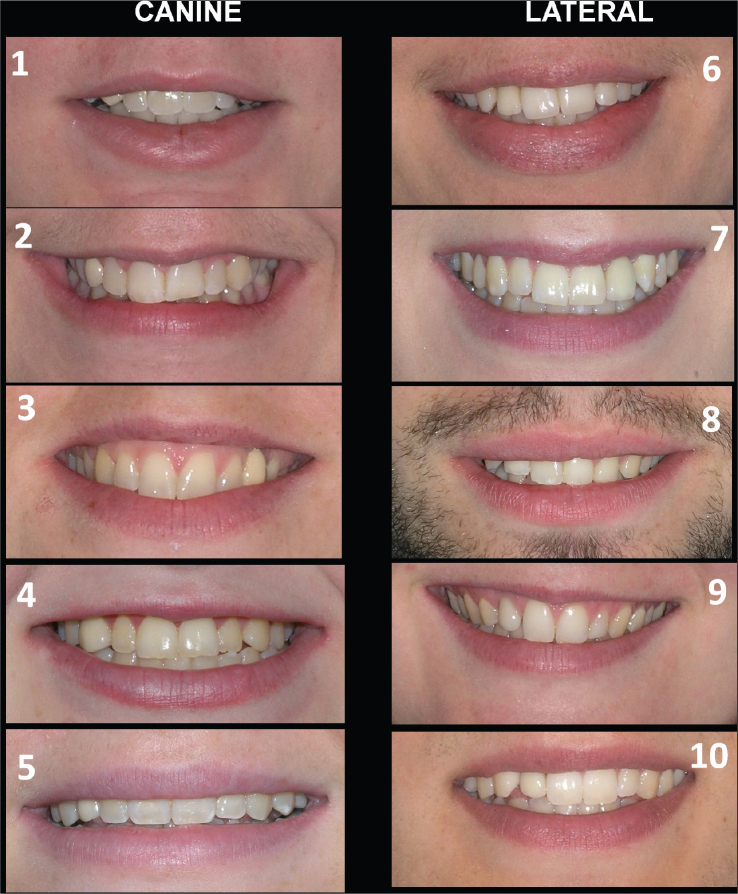
Smile photographs of cases where laypersons and orthodontists were asked to identify the artificial tooth. Implant-supported lateral crown on the right side is seen in cases #6, 8, 9, 10, and on the left side in case #7. Implant-supported canine crown on right side is seen in cases #1, 2, 4, 5, and on the left side in case #3. Highest score for orthodontists to correctly identify the implant-supported crown was seen for cases #2 and 3 (99%) and lowest score for case #8 (45%), while the laypersons showed the highest score to correctly identify the implant-supported crown for case #8 (92%) and lowest score for case #6 (8%).

Correct identification of the ISC was made by the respondents in 69% of the cases. The respondents identified correctly the ISC-L in 61% and ISC-C in 74% of the cases (*p* < 0.001). Orthodontists identified the ISC more correctly than laypersons (89% and 50%, respectively, *p* < 0.001) when viewing the smile photographs. Orthodontists more correctly identified the ISC-L, compared to the laypersons (83% and 41%, respectively, *p* < 0.001). Orthodontists more correctly identified the ISC-C, compared to laypersons (94% and 56%, respectively, *p* < 0.001) (Figure 1).

### Assessment of aesthetic parameters of implant-supported crowns

Of the ISCs, orthodontists rated higher proportions of acceptance for the aesthetic parameters; CI, SI, and GCI, each parameter individually, and all parameters considered as one, in favour for ISC-L, in comparison to ISC-C (*p* < 0.001). In addition, more ISC-C were assessed as having none of the aesthetic parameters as acceptable, compared to ISC-L (*p* < 0.001) (Table 1). Of the ISCs, laypersons rated higher proportions of acceptance of CI (*p* < 0.001) and GCI (*p* < 0.001) for the ISC-L, while SI was more favoured for the ISC-C (*p* = 0.05). When all aesthetic parameters were compared together, the ISC-L was assessed more acceptable than the ISC-C (*p* < 0.001) (Table 1).

**Table 1:** Numbers and percentage (in brackets) of acceptable proportions of the aesthetic parameters of implant-supported crowns, rated by orthodontists and laypersons. The responses ‘bad’ and ‘less good’ were grouped and represented as ‘non-acceptance of aesthetics’, and the alternatives ‘acceptable’, ‘good’ and ‘excellent’ were grouped and represented as ‘acceptance of aesthetics’. Chi-2 was calculated for acceptance/non-acceptance and lateral/canine for orthodontists and laypersons, respectively. *p*-values * *p* < 0.05, *** *p* < 0.001, ns: non-significant.

Acceptance of aesthetic parameters	Orthodontists			Laypersons		
	Lateral Implant supported crown *N* = 920	Canine Implant supported crown *N* = 920	*p*	Lateral Implant supported crown *N* = 1,035	Canine Implant supported crown *N* = 1,035	*p*
Colour implant (CI)	447 (49)	269 (29)	***	829 (80)	743 (72)	***
Shape implant (SI)	479 (52)	294 (32)	***	736 (71)	781 (75)	*
Gingival colour implant (GCI)	494 (54)	344 (41)	***	741 (72)	551 (43)	***
All parameters (CI, SI and GCI) None of the parameters (CI, SI and GCI)	215 (23) 194 (21)	90 (10) 410 (45)	*** ***	556 (54) 90 (9)	418 (40) 102 (10)	*** NS

**Figure 2:**
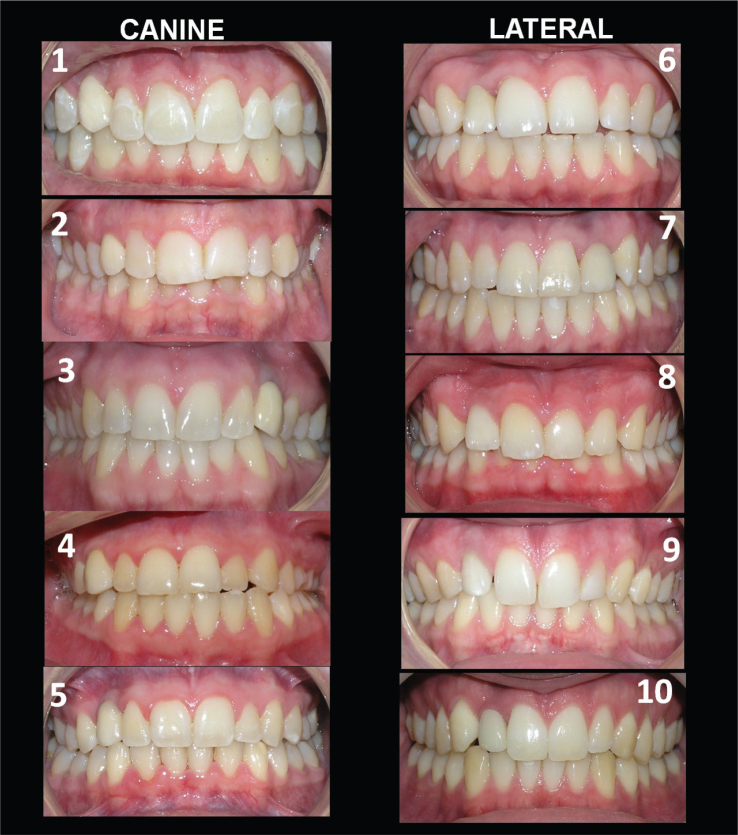
Intraoral photos of all cases where laypersons and orthodontists were asked to rate the aesthetic parameters: colour of tooth, shape of tooth and gingival colour of the implant-supported crown, in comparison to the contralateral tooth. The implant-supported lateral crown on the right side is seen in cases #6, 8, 9, 10, and on the left side in case #7. The implant-supported canine crown on the right side is seen in cases #1, 2, 4, 5, and on the left side in case #3.

Assessing the cases individually showed that five cases (case # 2, 4, 7, 9 and 10) were rated to be acceptable, when all three aesthetic parameters were rated as acceptable, in ≥ 50% of the laypersons. Most of the orthodontists’ responses showed that no cases were rated to be acceptable when viewing the three aesthetic parameters as one. The total acceptance was seen in larger proportions by the laypersons, compared to orthodontists, in all cases (*p* < 0.001) except for case 6 (*p* = 0.212) (Table 2, Figure 2).

**Table 2:** Proportion of acceptance for all three aesthetic parameters considered as one unit of correctly identified implant-supported crowns, data presented for the cases as rated by orthodontists and laypersons. Chi-2 was calculated for acceptance/non-acceptance and orthodontists/laypersons. ***p < 0.001, ns = non-significant.

Case	Orthodontists Acceptance of all three aesthetic parameters for implant-supported crowns *N* = 308 (17%)	Laypersons Acceptance of all three aesthetic parameters for implant-supported crowns *N* = 974 (47%)	*p*
1	37 (20%)	80 (39%)	***
2	33 (18%)	141 (68%)	***
3	0 (0%)	42 (20%)	***
4	14 (8%)	116 (56%)	***
5	6 (3%)	39 (19%)	***
6	88 (48%)	86 (42%)	NS
7	24 (13%)	103 (50%)	***
8	44 (24%)	97 (47%)	***
9	21 (11%)	138 (67%)	***
10	41 (22%)	132 (64%)	***

In 24% of the cases with ISC-L, the orthodontists rated acceptance for all three aesthetic parameters, compared to 10% for the ISC-C. The laypersons assessed 54% of the ISC-L as acceptable, and 40% of the ISC-C.

### General importance of the aesthetic parameters

The orthodontists and laypersons rated the general importance of the aesthetic parameters. The colour of the tooth was valued higher by orthodontists compared to laypersons (*p* < 0.001). The tooth shape was rated higher by orthodontists in comparison to by laypersons (*p* < 0.001). The gingival colour was evaluated higher by orthodontists related to laypersons’ rating (*p* < 0.001) (Table 3).

**Table 3:** Descriptive statistics of general judgement of importance of the aesthetic parameters tooth colour, tooth shape, and gingival colour evaluated by orthodontists and laypersons, using a VAS-scale ranging 0–10. 0: Parameter being not of importance, 10: Parameter being of the greatest importance. Independent T-test for the differences between orthodontists and laypersons of each parameter.

Parameter	Orthodontists			Laypersons			*T*-test
	Mean	SD	CI 95%	Mean	SD	CI 95%	*p*
**Tooth colour**	9.05	1.017	8.91–9.20	8.03	1.494	7.82–8.23	> 0.001
**Tooth shape**	8.84	1.184	8.67–9.01	8.00	1.743	7.76–8.24	> 0.001
**Gingival colour**	8.01	1.676	7.77–8.25	7.16	2.244	6.85–7.47	> 0.001

**Figure 3:**
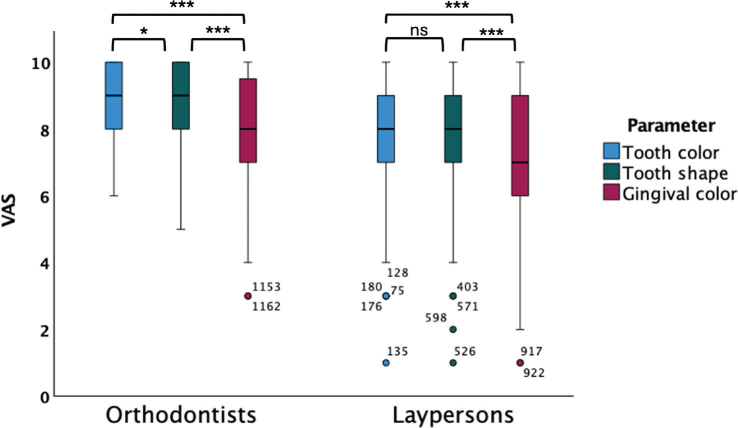
Boxplot of the general judgement of importance of the aesthetic parameters colour, shape of the implant-supported crown, and gingival colour evaluated by orthodontists and laypersons, using a VAS-scale ranging 0–10. 0: Parameter being not of importance, 10: Parameter being of the greatest importance. Orthodontists ranked tooth colour as being the most important parameter in relation to tooth shape (*p* < 0.05) and gingival colour (*p* < 0.001). Tooth shape was ranked being more important than gingival colour (*p* < 0.001). Laypersons ranked tooth colour and tooth shape being more important than gingival colour (*p* < 0.001). Comparisons of mean performed by independent-samples *t*-test, *p < 0.05, ***p < 0.001, ns = non-significant.

Gingival colour was considered being the least important for both the groups. The most important parameter ranked by the orthodontists was tooth colour in relation to tooth shape (*p* > 0.05) and gingival colour (*p* > 0.001). Laypersons ranked tooth colour and shape being more important than gingival colour (*p* > 0.001), with no difference in ranking between tooth colour and shape (ns) (Figure 3).

## Discussion

Several studies suggest that laypersons prefer orthodontic space closure with canine substitution over implant treatment [7, 35, 39]. However, there are cases when the conditions are favourable for space creation for an ISC. The results gained from the present study show that ISC-L is preferable.

This study was justified as there are only few studies in the literature reporting aesthetic outcome of treatments replacing a missing maxillary canine [9, 40]. A strength of the study is the high number of respondents, making the response rate considered as high. The response rate for the orthodontists is similar to another study, whose survey was distributed through the Swedish Orthodontic Society [41]. To answer the questions of the survey, Likert-scale options were given, as well as one question using the VAS-scale; both commonly used methods to assess facial attractiveness [42, 43]. Other studies have conducted similar surveys with photographs of the mouth [35, 44, 45]. The present study contained 10 cases and a high number of respondents to maintain focus. Nevertheless, there are studies with more cases and fewer participants estimating the esthetical outcome [9, 46, 47].

The results from this study showed that ISC-C is more frequently discerned than ISC-L by the respondents. Furthermore, the orthodontists distinguished ISCs to a higher degree than laypersons. This finding is not surprising as orthodontist are less tolerant to aesthetic discrepancies than laypersons [39, 48]. Additionally, the results showed that ISC-L is more aesthetically acceptable than ISC-C. This finding was supported by both laypersons and orthodontists. Differences in morphologies may suggest why the aesthetics of the ISC-L scored higher than ISC-C. The size and form of the maxillary anterior teeth are important for dental and facial aesthetics. The most harmonious smile has been shown to be the one with the golden proportions; the width of the anterior tooth is 60% the width of the adjacent tooth, thus the lateral incisors should be 60% the width of the central incisors, and the canine 60% that of the lateral incisors [49]. The canine is a larger tooth as well as being placed in the curvature of the maxillary arch, making it more prominent than the lateral incisor [49, 50]. According to a systematic review, laypersons preferred flat, canine viewing photographs of male models, while no preferences were found for female models. Laypersons also preferred small teeth in images of female models and large teeth in images of male models [49]. It is well-known that dental aesthetics is important for the attractiveness of a face. Different factors affect the appearance of a smile such as tooth colour, shape, size, upper lip position, and gingival display [14]. These factors act together as one unit to produce the final aesthetic effect. Tooth colour is one of the most important factors that influences the perception of a smile [51]. In this study, when asking about tooth aesthetics in general, that is not considering ISCs, both colour and shape were considered more important than gingival colour. This is in accordance with another Swedish study showing that 18- to 19-year-old patients are more dissatisfied with the colour of the anterior teeth than irregularity, spacing, or increased overjet [52].

Viewing the cases, laypersons were more accepting to all aesthetic parameters, except for the GCI of the ISC-C. The proportion of laypersons rating acceptance for GCI of the ISC-C were equal to the proportion of orthodontists. For reasons unexplained, laypersons critically assessed the GCI of the ISC-C. Meijndert et al. also found that the soft tissue surrounding the implant crown was rated both by laypersons and dentists as less aesthetically satisfying, compared to the crown design [23]. Furthermore, aesthetic outcome of the gingiva is reported as being less appealing in implant treatments in cases with agenesis of laterals compared to space closure [7, 39]. Discoloured labial mucosa around the implant and the adjacent gingiva, papilla defect, and infra-occlusion are few of the long-term, negative observations found in patients treated with ISCs [7, 39, 53].

Most of the laypersons scored that ISC-L was acceptable in the three-evaluated aesthetic parameter – crown colour, shape, and gingival colour. A majority outcome was not found for the ISC-C rated by laypersons, or for the ISC in either position, when orthodontists evaluated. Nevertheless, acceptance to all aesthetic parameters may be considered a high requirement. However, considering all parameters, orthodontists found ISC-L to be more acceptable in comparison to the ISC-C. Orthodontists were more critical in judging the aesthetic outcome, compared to laypersons. These results were expected since orthodontists are analytical when observing aesthetics, due to specialist training [26, 39]. Therefore, the threshold to score an asymmetry as aesthetically unattractive is lower in orthodontists than in laypersons [28].

Implant is preferred to be installed when the growth of the maxilla has ceased, approximately around 20 years of age [54, 55]. Therefore, laypersons aged 20–25 years were asked to participate in this study, to represent the patients the cases were built upon. Young laypersons have high demands and are critical when assessing aesthetic parameters [16, 24]. In addition, satisfactory aesthetic outcome is influenced by society, media, and culture, from where social norms and ideals are created [56]. This is especially true in today’s society where social media have a big influence. Dentistry today is focussed on person-centred care, where the patient’s well-being is placed in the center of treatment-planning, to achieve optimal oral health care [57]. For future research projects, it would be valuable to study the long-term satisfaction, by both practitioners and patients, of ISCs in the aesthetic region.

## Limitations

The survey was long and took considerable time to answer. This could have had a ‘straight lining’ effect on the answers toward the end of the survey, since laypersons are inexperienced in viewing intraoral photographs. A straight lining effect may be seen when the respondents select the same response every time. This may be due to boredom, stress, or no longer motivated to respond to the survey [58]. The generalisability of the included cases might not be representative for all cases with ISCs on the lateral and canine position. However, since the number of participants was high, the results can be generalised for the studied population.

One limitation of the study might be the quality of the clinical photos the survey is based on, though the survey was digital and could be answered on any digital device that is smartphone or computer. The devise used contributes to differences when viewing. On the other hand, digital viewing is considered better than viewing photos on paper. In addition, another restriction of the study is the distribution of the survey, many of the participants (laypersons) were asked to scan a QR-code to answer the survey, while the orthodontists received the survey via e-mail. The varying size of screen when viewing photos, might be reflected in the responses.

## Conclusions

An ISC placed on the lateral position is more aesthetically preferable than on the canine position, though orthodontists were more critical than laypersons. The colour and the shape of the tooth, in general, is rated as being more aesthetically important than the gingival colour by laypersons and orthodontists. These findings should be taken into consideration when planning ISCs in the aesthetic region.
